# Melanopsin photoreception differentially modulates rod-mediated and cone-mediated human temporal vision

**DOI:** 10.1016/j.isci.2022.104529

**Published:** 2022-06-03

**Authors:** Samir Uprety, Prakash Adhikari, Beatrix Feigl, Andrew J. Zele

**Affiliations:** 1 Centre for Vision and Eye Research, Queensland University of Technology (QUT), Brisbane, QLD 4059, Australia; 2 School of Optometry and Vision Science, Queensland University of Technology (QUT), Brisbane, QLD 4059, Australia; 3 School of Biomedical Sciences, Queensland University of Technology (QUT), Brisbane, QLD 4059, Australia; 4 Queensland Eye Institute, Brisbane, QLD 4101, Australia

**Keywords:** Applied sensory psychophysics, Cellular neuroscience, Sensory neuroscience

## Abstract

To evaluate the nature of interactions between visual pathways transmitting the slower melanopsin and faster rod and cone signals, we implement a temporal phase summation paradigm in human observers using photoreceptor-directed stimuli. We show that melanopsin stimulation interacts with and alters both rod-mediated and cone-mediated vision regardless of whether it is perceptually visible or not. Melanopsin-rod interactions result in either inhibitory or facilitatory summation depending on the temporal frequency and photoreceptor pathway contrast sensitivity. Moreover, by isolating rod vision, we reveal a bipartite intensity response property of the rod pathway in photopic lighting that extends its operational range at lower frequencies to beyond its classic saturation limits but at the expense of attenuating sensitivity at higher frequencies. In comparison, melanopsin-cone interactions always lead to facilitation. These interactions can be described by linear or probability summations and potentially involve multiple intraretinal and visual cortical pathways to set human visual contrast sensitivity.

## Highlights

• Melanopsin ipRGCs support vision independent of the rod and cone signals
• Rod pathways mediate robust visual responses in daylight
• Temporal contrast sensitivity is contingent on the melanopsin excitation level
• Visual performance is collectively regulated by melanopsin, rod and cone pathways

## Graphical abstract

**Figure:**
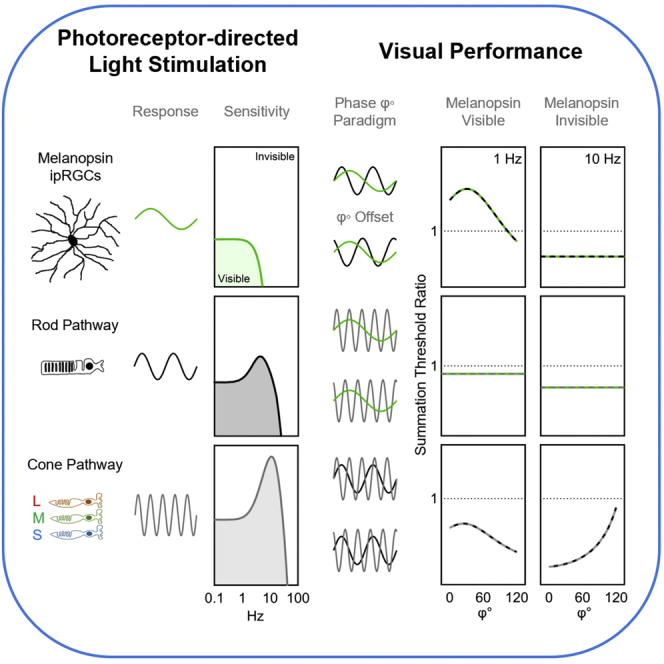

## Introduction

The temporal response properties of the cone pathway are faster than for the rod pathway (Lee et al., 1990), with the response increasing with higher retinal illumination (Conner, 1982; de Lange, 1954; Kelly, 1961). These temporal response differences lead to rod-cone interactions (Buck, 1985; Cao and Lu, 2012; Cao et al., 2006; Naarendorp et al., 1996; Sharpe et al., 1989b; Sun et al., 2001; Van den Berg and Spekreijse, 1977; Zele et al., 2008; Zele et al., 2012) and cone-cone interactions (Eisner, 1995; Kremers et al., 1993; Sun et al., 2001) that alter visual sensitivity and modify the perceptual experience. It is the sharing of neural pathways that supports these interactions (reviewed in Lee 2011; Thoreson and Dacey 2019). Such interactions are not yet known for the melanopsin pathway.

Intrinsically photosensitive Retinal Ganglion Cells (ipRGCs or giant sparse ganglion cells) provide a shared pathway for their intrinsic melanopsin response and extrinsic rod and cone signals in nonhuman primates (Dacey et al., 2005; Grünert et al., 2011; Jusuf et al., 2007; Patterson et al., 2020) and humans (Liao et al., 2016; Nasir-Ahmad et al., 2019). Recordings from nonhuman primate retinae show that intrinsic melanopsin responses have longer implicit times to peak spike frequency than do cone and rod responses (Dacey et al., 2005). The critical flicker frequency (CFF) for melanopsin-mediated vision is also lower than for rod-mediated or cone-mediated human vision (Zele et al., 2018b). Here, we explore the effect of differences in the melanopsin, rod and cone pathway temporal and adaptation characteristics on interactions affecting visual contrast sensitivity.

**Figure 1:**
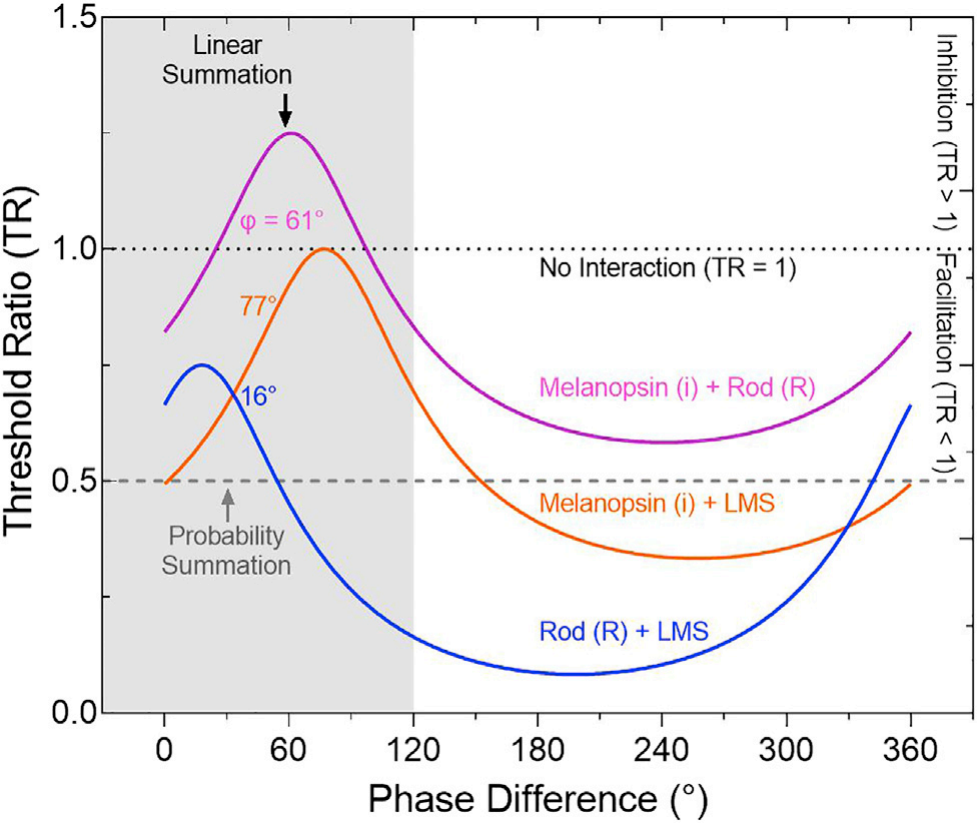
Exemplar model predictions for the phase summation paradigm Combined photoreceptor to individual photoreceptor-directed threshold ratios (TR) for three photoreceptor-directed combination stimuli (1 Hz; i + R, i + LMS, R + LMS) are shown as a function of their relative phase difference (degrees). If there is no effect of the relative phase of the combined stimuli on the visual threshold, then the TRs will follow a straight line (no interaction: TR = 1). The presence of an interaction causes inhibition (TR > 1) or facilitation (TR < 1). Probability summation (TR = 0.5; Equation 1) can occur when threshold changes are independent of phase. If the interaction is phase-dependent, the model predicts linear summation (curved lines at three different phase delays, φ; Equation 2). The shaded region covers the experimental phase differences measurable within the instrument gamut.

For vision, higher levels of melanopsin excitation can improve cone-mediated contrast discrimination (Zele et al., 2019b), with both the melanopsin and cone pathways supporting brightness estimation (Brown et al., 2012; DeLawyer et al., 2020; Zele et al., 2018a). For pupil control, the rod or cone interactions with melanopsin follow linear summation within ipRGCs (Barrionuevo and Cao, 2016; Barrionuevo et al., 2014; Gooley et al., 2012; McDougal and Gamlin, 2010; Zele et al., 2019a). A retinal source for the photoreceptor interactions is evidenced by the dependence of the photopic cone-mediated b-wave amplitude of the human electroretinogram (ERG) on the melanopsin excitation level (Adhikari et al., 2019; Fukuda et al., 2010; Hankins and Lucas, 2002). Although the intraretinal networks are the same for the pupil and vision pathways, primate ipRGCs singularly transmit the intrinsic melanopsin signal in addition to extrinsic outer retinal photoreceptor signals for pupil control (Gamlin et al., 2007; Ostrin et al., 2018), with rod and cone vision mediated via the canonical retinogeniculate pathways (Lee et al., 2010). Any such vision-dependent interactions between the melanopsin and rod-cone signals could therefore be supported through networks involving amacrine cells (Marshak et al., 2015; Patterson et al., 2020) that can modulate intraretinal ipRGC signals to rods and cones in nonhuman primates (Jusuf et al., 2007; Liao et al., 2016; Patterson et al., 2020) and mice (Østergaard et al., 2007; Zhang et al., 2008; Zhao et al., 2014). Alternatively, a cortical detection site could provide a locus of the interactions. Here, we implement a phase-summation paradigm (Lee et al., 1990; Smith et al., 1992; Sun et al., 2001) to determine the nature of the melanopsin and rod and cone photoreceptor pathway interactions (Figure 1).

We conduct our experiments in mesopic and photopic illumination where the melanopsin, rod and cone pathways are active. Rods are traditionally thought to saturate in daylight photopic illumination (∼300 cd m^−2^, 5000 Sc Td, 2000 Ph Td) (Aguilar and Stiles, 1954). However, this loss of rod pathway contrast sensitivity is not because of photopigment bleaching (>94% rhodopsin availability at 300 cd m^−2^) (Rushton and Powell, 1972; Thomas and Lamb, 1999) but instead involves interactions with cone signals at post-receptoral sites (Rushton and Westheimer, 1962; Shapiro, 2002; Sharpe et al., 1989a). As such, modern measurements show that in higher photopic illumination, the rod pathway can mediate visual contrast sensitivity (Hess and Nordby, 1986; Shapiro, 2002; Sharpe et al., 1989a), input to the pupil control pathway (Adhikari et al., 2016; Barrionuevo and Cao, 2016; McDougal and Gamlin, 2010), and generate robust ERG responses in humans (Kremers et al., 2009; Maguire et al., 2016), mice (Tikidji-Hamburyan et al., 2017), and rod-only skate retina (Hu et al., 2021). Rod pathways can also drive circadian photoentrainment in mice during daytime (Altimus et al., 2010; Güler et al., 2008). The transition illumination to rod saturation is therefore complexly dependent on the viewing and measurement conditions. Given the peak spectral sensitivities of melanopsin and rhodopsin are in the shorter-wavelength region of the visible spectrum, silent-substitution methods developed to independently modulate melanopsin excitation must establish the interaction type and tolerance limits to rod signaling with the measured melanopsin function. Therefore, we evaluate whether rods escape saturation at high photopic illumination and as part of this analysis, we quantify the magnitude of rod intrusion that can be tolerated in melanopsin-directed stimuli without affecting the characteristic melanopsin temporal contrast response. We report that melanopsin stimulation interacts with both the rod and cone pathways to alter human temporal contrast sensitivity.

## Results

### Precisely controlled lights reveal the relative photoreceptor inputs to human temporal vision

**Figure 2:**
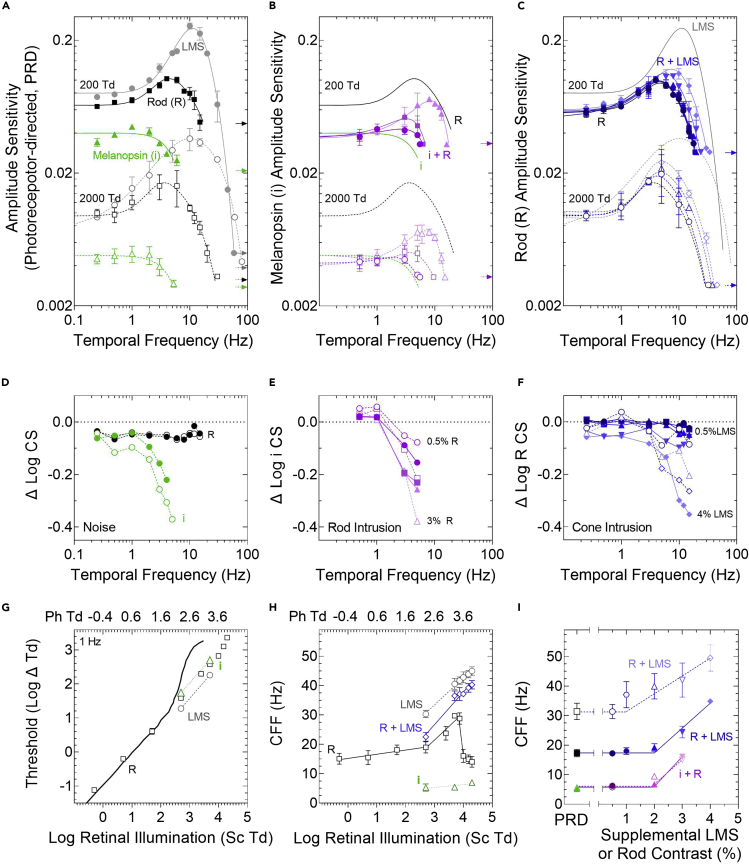
Photoreceptor-directed temporal response characteristics in mesopic and photopic lighting (A) Amplitude sensitivity (mean ± SEM, n = 3 observers) as a function of temporal frequency (symbols) is described by best-fitting difference of Gaussians models (curved lines) at mesopic (200 Td, filled symbols, solid lines) and photopic illumination (2000 Td, unfilled symbols, dashed lines). Temporal sensitivity is low pass for melanopsin-directed (green triangles) and band-pass for both rod-directed (black squares) and LMS-cone-directed stimuli (gray circles). Arrows denote the instrument gamut for each condition. (B) Melanopsin-mediated visual function (green line from (A)) is enhanced with subthreshold, supplemental rod contrast (magenta symbols and lines; + 0.5% Michelson rod contrast, circles; + 2%, squares; + 3%, triangles). (C) Rod-mediated visual function shifts toward the cone sensitivity function with supplemental cone contrast (blue symbols and lines; + 0.5% Michelson LMS cone contrast, circles; + 1%, squares; + 2%, triangles; + 3%, inverted triangles; + 4%, diamonds). (D and F) Application of temporal white noise desensitizes the cone intrusions at higher frequencies in melanopsin-directed stimuli. The change (Δ log CS) in melanopsin-directed or rod-directed contrast sensitivity indicates that (E) supplemental rod-contrast or (F) cone-contrast in the stimuli can facilitate temporal sensitivity at frequencies >1 Hz. (G) The threshold versus intensity (TVI) response function for rod-directed stimuli (1 Hz, squares) is more sensitive than, and deviates from the classic Aguilar and Stiles (1954) model at illuminations >498 Sc Td (200 Ph Td) (solid black line; −0.12 vertical adjustment to account for differences in spatiotemporal summation). In photopic illuminations, the rod-pathway has lower contrast sensitivity than the cone pathway (LMS; gray circles) but higher sensitivity than the melanopsin-pathway (i; green triangles). (H) The rod-directed critical flicker frequency (CFF) (black squares) is attenuated by illuminations >7470 Sc Td (3000 Ph Td). The cone CFF increases with light level according to the Ferry-Porter law (gray circles). Rod-cone interactions (Rod +3% supplemental LMS-cone contrast; blue diamonds) suppress the LMS-cone CFF to a level between the rod-directed and cone-directed maximum temporal resolution. The melanopsin CFF is invariant with changes in retinal illumination (green triangles). (I) The CFF of the three photoreceptor-directed (PRD) conditions increase with intrusion from supplemental photoreceptor contrast at two light levels (200 Td, darker lines and symbols; 2000 Td, lighter lines and symbols).

In both mesopic (200 Td) and photopic (2000 Td) illumination, absolute amplitude sensitivity to the melanopsin-directed temporal modulation is low pass (Figure 2A, green) with a critical flicker frequency (CFF) at 5.7 ± 1.1 Hz (mean ± standard error of the mean, SEM; n = 3 observers). Contrast sensitivity was converted to absolute amplitude sensitivity (Kelly, 1961) to evaluate at lower frequencies, the ratio of the required change in stimulus contrast with variation in adaptation light level (i.e., Weberian behavior, W = ΔI/I), and at higher frequencies, the change in CFF with adaptation level (i.e., the Ferry-Porter law). A Weber-like adaptation response is evident for melanopsin-directed stimulation at low frequencies with transition between 200 Td and 2000 Td illumination (Figure 2G, W = 0.96). For frequencies ≤ 1Hz, which are in the range of its peak temporal sensitivity, melanopsin is robust to rhodopsin intrusion (i.e., supplemental rod contrast) in the stimulus (Figures 2B and 2E). For frequencies > 1Hz, the melanopsin-directed TCSF significantly shifts toward the rod pathway function with rod intrusion ≥3% Michelson contrast. When compared to the melanopsin-directed stimuli, supplemental rod contrast significantly affects melanopsin sensitivity (RM-ANOVA; 200 Td, F_3,24_ = 16.16, p = 0.002, *Mauchly’s Test: χ*^*2*^_*5*_ *= 8.46,* p *= 0.132*; 2000 Td, F_3,24_ = 19.13, p = 0.002, *χ*^*2*^_*5*_ *= 9.37,* p *= 0.095*). The pairwise statistical comparisons identified a significant effect of ≥3% supplemental Michelson rod contrast (200 Td, p = 0.01; 2000 Td, p = 0.016) and nonsignificant effects at lower supplemental rod contrasts (for 0.5%: 200 Td, p = 0.057; 2000 Td, p = 0.19 and for 2%: 200 Td, p = 0.296; 2000 Td, p = 0.9). Strikingly, at frequencies beyond the temporal resolution of melanopsin vision (>5 Hz), melanopsin-directed modulations become visible with subthreshold supplemental rod contrast (3% Michelson contrast) indicating a subthreshold summation between melanopsin and rod signals (Figures 2B and 2E). The melanopsin-directed CFF is independent of retinal illumination up to 8000 Td (Figures 2A and 2H) and significantly increases with ≥3% supplemental rod contrast (Figure 2I, magenta) (RM-ANOVA; main effect of rod contrast; 200 Td, F_3,6_ = 315.35, p = 0.000005, *χ*^*2*^_*5*_ *= 10.81,* p *= 0.05*; 2000 Td, F_3,6_ = 181.78, p = 0.000003, *χ*^*2*^_*5*_ *= 10.29,* p *= 0.06*). The criterion supplemental rod intrusion (≥3% contrast) required to shift the melanopsin TCSF is greater than the theoretical rod intrusion present in the melanopsin-directed stimuli (≤0.3% rod contrast; see STAR Methods).

In mesopic and photopic illumination, the rod-directed response is band pass, with a contrast sensitivity and temporal resolution higher than the melanopsin pathway (Figure 2A, black). The application of TWN does not have a measurable effect on rod-directed responses (Figure 2D, black), indicating no cone intrusion. At low frequencies (1 Hz), a sub-Weber adaptation response is evident with transition between 0.2 Td and 200 Td (W = 0.84) as per Aguilar and Stiles (1954) (Figure 2G, model, vertically adjusted by −0.012 to account for higher visual sensitivity with flickering stimuli and a larger stimulus area). Rod thresholds remain measurable up to the instrument gamut limit (8000 Td with a maximum 11% rod contrast) (Figure 2G, black squares) with a Weber-like slope (W = 1.05) between 200 and 3000 Td that begins to increase (W = 1.27) beyond 3000 Td and indicates reduced contrast sensitivity. At higher temporal frequencies, a different pattern is found; the rod-directed CFF increases through low photopic (∼18 Hz) to a peak in high photopic illumination (∼27 Hz; Figure 2H, black squares). Higher photopic illuminations attenuate (3000–8000 Td, ∼7470–19920 Sc Td) but do not saturate the rod CFF, returning the response to its mesopic performance values (Figure 2G, black squares). For frequencies <3 Hz, rhodopsin is robust to cone intrusion (supplemental cone contrast) in the stimulus (Figures 2C and 2F blue). For frequencies > 3Hz, the rod-directed TCSF shifts toward the cone pathway function with cone intrusion levels ≥3% Michelson contrast, with the TCSF peak increasing from 4 to 9 Hz at 200 Td and from 3 to 8 Hz at 2000 Td, and the CFF increasing from 17 to 38 Hz at 200 Td and from 31 to 50 Hz at 2000 Td, approaching the cone-mediated TCSF. When compared to the rod-directed stimuli, supplemental cone contrast significantly affects rod pathway contrast sensitivity (RM-ANOVA; 200 Td, F_5,65_ = 18.91, p = 0.000014, *χ*^*2*^_*14*_ *= 22.49,* p *= 0.69*; 2000 Td, F_3,30_ = 16.48, p = 0.00002, *χ*^*2*^_*5*_ *= 9.82,* p *= 0.08*). The pairwise statistical comparisons identified a significant effect with ≥3% supplemental cone contrast (200 Td, p = 0.000014; 2000 Td, p = 0.00002) and a nonsignificant effect of lower supplemental cone contrasts (for 0.5%: 200 Td, p = 0.99; 2000 Td, p = 0.16, for 1%: 200 Td, p = 0.93, and for 2%: 200 Td, p = 0.39; 2000 Td, p = 0.52). The criterion cone intrusion (≥3%) required to shift the rod TCSF is greater than the theoretical cone intrusion present in the rod-directed stimuli (≤1.5% cone contrast; see STAR Methods). In general, the progressively higher supplemental cone contrasts cause a linear increase in the rod-directed CFF (Figure 2I blue). Although the supplemental cone contrast (3%) increases the rod-directed CFF (Figure 2H, blue), suppressive rod-cone interactions prevent it from reaching the levels of the cone-pathway (Figure 2H, gray circles).

The LMS-cone-directed absolute sensitivity is band pass (Figure 2A, gray), with contrast sensitivity and temporal resolution higher than rods, a Weberian behavior at low frequencies (W = 0.98) (Figure 2G, gray circles), and a Ferry-Porter behavior at high frequencies (Figures 2H and 2I). That our data demonstrate the melanopsin-directed TCSF peaks at low frequencies (≤1 Hz) and is imperceptible beyond ∼6 Hz, we then evaluated the melanopsin and rod-cone interaction at a frequency near the peak of the melanopsin TCSF (1 Hz) and at a frequency beyond its visual temporal resolution (10 Hz).

### Differential phase delays between photoreceptor classes are a driver of temporal sensitivity

**Figure 3:**
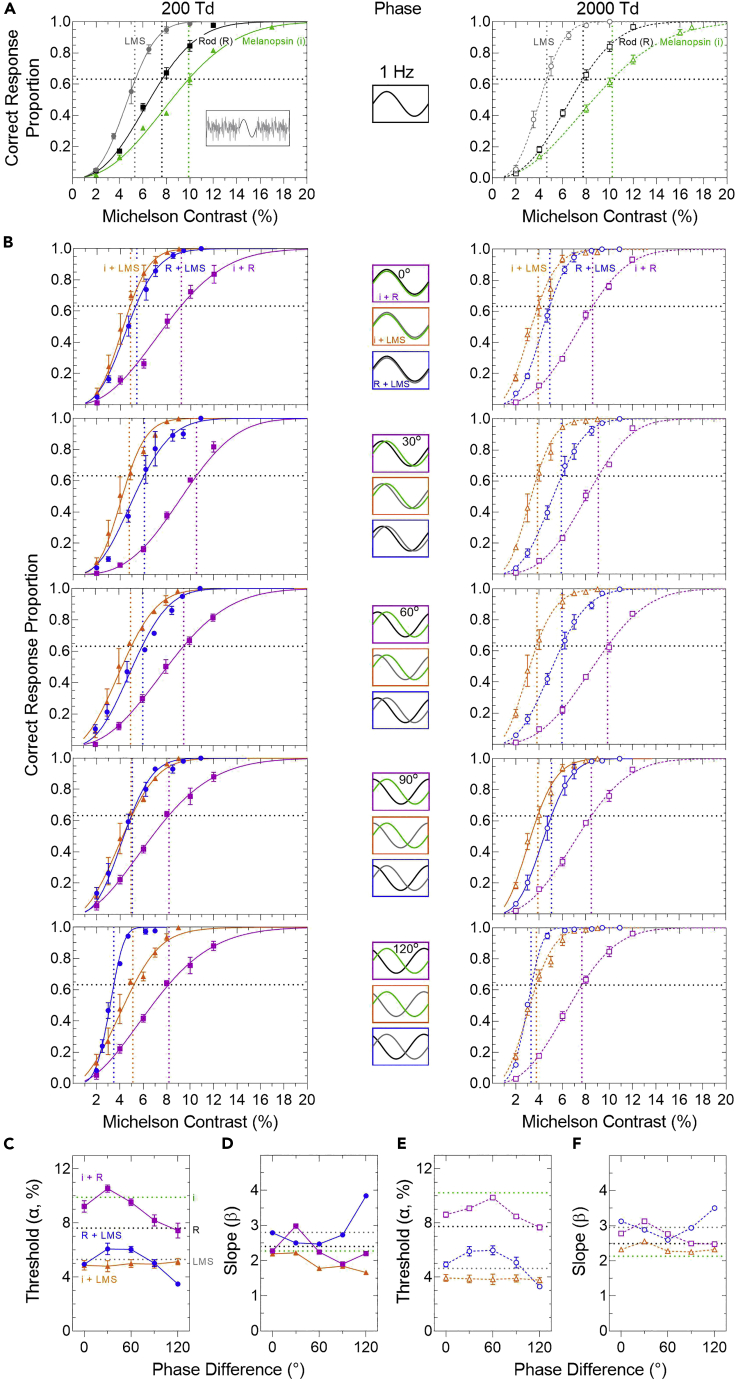
Phase-dependant changes in the 1Hz combined photoreceptor-directed contrast responses in mesopic and photopic lighting (A) Psychometric functions for melanopsin (i)-directed stimuli (triangles, Weibull models in green) show higher thresholds (α, vertical dotted lines intersecting at 63.21% yes responses, horizontal dotted line) than for rod (R)-directed (squares, black lines) and LMS cone-directed stimuli (circles, gray lines) at 200 Td (filled symbols, solid lines) and 2000 Td (unfilled symbols, dashed lines). Data show the mean ± SEM (n = 3 observers). (B) Psychometric functions for the combination i + R-directed (magenta squares), i + LMS-directed (orange triangle) and the R + LMS-directed stimuli (blue circles) as a function of their relative phase difference (0–120°). The Michelson contrast on the abscissa represents the rod contrast for the i + R and R + LMS combinations and represents the LMS-cone contrast for the i + LMS combination. (C and E) The combination thresholds (α) are phase dependent with i + R-directed (magenta squares) and R + LMS-directed stimuli (blue circles) but independent of phase with i + LMS-directed stimuli (orange triangles). Combination thresholds below the respective dotted horizontal line indicate facilitation whereas those above the line indicate inhibition. (D and F) Slope (β) of the psychometric functions for the relative phase differences of the combined stimuli with reference to their individual photoreceptor-directed metrics (horizontal dotted lines).

**Figure 4:**
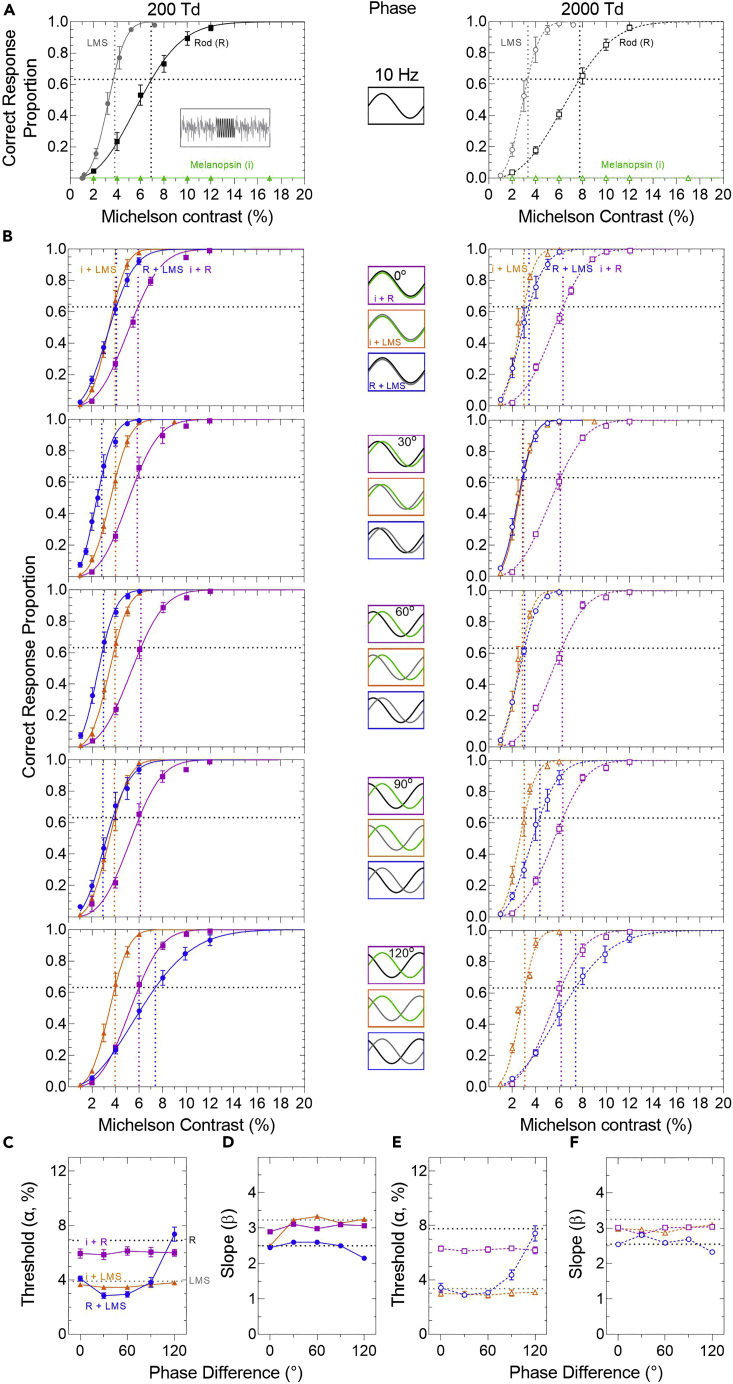
Phase-dependent changes in the 10 Hz combined photoreceptor-directed contrast responses in mesopic and photopic lighting (A) Psychometric functions for melanopsin (i)-directed stimuli (triangles, Weibull models in green) show higher thresholds (α, vertical dotted lines intersecting at 63.21% yes responses, horizontal dotted line) than for rod (R)-directed (squares, black lines) and LMS cone-directed stimuli (circles, gray lines) at 200 Td (filled symbols, solid lines) and 2000 Td (unfilled symbols, dashed lines). Data show the mean ± SEM (n = 3 observers). (B) Psychometric functions for the combination i + R-directed (magenta squares), i + LMS-directed (orange triangle) and the R + LMS-directed stimuli (blue circles) as a function of their relative phase difference (0–120°). The Michelson contrast on the abscissa represents the rod contrast for the i + R and R + LMS combinations and represents the LMS-cone contrast for the i + LMS combination. (C and E) The combination thresholds (α) are phase dependent with R + LMS-directed stimuli (blue circles) but independent of phase with i + R-directed (magenta squares) and i + LMS-directed stimuli (orange triangles). Combination thresholds below the respective dotted horizontal line indicate facilitation, whereas those above the line indicate inhibition. (D and F) Slope (β) of the psychometric functions for the relative phase differences of the combined stimuli with reference to their individual photoreceptor-directed metrics (horizontal dotted lines).

The threshold (α) and slope (β) parameters for the three photoreceptor-directed stimulus conditions were estimated using the Weibull model to fit the measured psychometric functions (Figure 3, Figure 4A and 4A; Table 1). The higher temporal frequency (10 Hz) is beyond the visual resolution limits of the melanopsin pathway (Figure 4A), whereas the lower frequency (1 Hz) is within the temporal resolution of the melanopsin and rod and cone pathways (Figure 3A). Melanopsin-directed thresholds (α) are always higher than rhodopsin-directed, which are higher than LMS cone-directed thresholds. Psychometric slopes (β) are always shallower for melanopsin-directed than rod-directed or cone-directed stimuli (Figure 3, Figure 4A and 4A). We find evidence of an interaction with a unique phase dependent change in threshold (α) and slope (β) for each of the three photoreceptor-interaction combinations when measured in a 1:1 threshold unit (Figure 3, Figure 4C–3F and 4C–4F) as described in the next section.

**Table 1:** Weibull psychometric function and summation model parameters for individual and combined photoreceptor-directed modulations

				Photoreceptor-directed stimuli (μ±SEM)			Combination stimuli (μ±SEM)		
				Melanopsin (i)	Rod (R)	LMS	i + R	i+ LMS	R + LMS
1 Hz	Weibull	200 Td	α	9.9±0.1	7.6±0.1	5.3±0.1	8.9±0.5	4.9±0.1	5.1±0.5
			β	2.2	2.4	2.8	2.3±0.2	1.9±0.1	2.8±0.2
		2000 Td	α	10.2±0.3	7.7±0.0	4.64±0.3	8.7±0.3	3.8	5.0±0.5
			β	2.1	2.5	2.9	2.7±0.1	2.3±0.1	3.0±0.1
	Summation	200 Td					i = 0.2±0.1 r = 1.0±0.1 φr-i = 30.4°	n = 0.9±0.0	r = 0.6±0.0 c = 1.8±0.1 φ_c-r_ = 24.7°
		2000 Td					i= 0.3 r = 1.2±0.1 φr-i = 45.7°	n = 0.8±0.0	r = 0.6±0.1 c = 1.9±0.1 φ_c-r_ = 28.1°
10 Hz	Weibull	200 Td	α		6.9±0.5	3.8±0.6	5.9±0.1	3.6±0.1	4.2±0.8
			β		2.5	3.2	3.0	3.0±0.1	2.4±0.1
		2000 Td	α		7.7±0.3	3.3±0.3	6.2	2.9±0.1	4.2±0.8
			β		2.5	3.2	2.9	3.2±0.2	2.5±0.1
	Summation	200 Td					n = 0.8±0.0	n = 0.9±0.0	r = 1.1±0.0 c = 2.3±0.0 φ_c-r_ = 163.4°
		2000 Td					n = 0.8±0.0	n = 0.8±0.0	r = 1.2±0.0 c = 2.5±0.1 φ_c-r_ = 170.0°

μ, mean; SEM, Standard Error of the Mean; α, threshold (% Michelson contrast); β, slope; *i*, melanopsin; *r*, rods; *c*, LMS-cones; φ, relative phase difference; *n*, probability summation index in linear units (= 20∗log_10_1/n dB).

At 1 Hz, the combined threshold (α) and slope (β) are dependent on the phase of the melanopsin + rod-directed modulation (Figure 3B), with the maximal elevation between 30° (200 Td; Figure 3C, magenta) and 60° (2000 Td; Figure 3E, magenta) where the psychometric slope is steepest (Figures 3D and 3F). The 1 Hz combined thresholds are generally higher than the rod-directed threshold (black dashed line, i.e., rod-pathway inhibition). At 10 Hz, the combined thresholds (Figures 4C and 4E) and slopes (Figures 4D and 4F) are independent of the phase of the melanopsin + rod-directed modulation at both illuminations. The 10 Hz combined thresholds are generally lower than the rod-directed threshold (i.e., rod-pathway facilitation).

At both temporal frequencies (1 and 10 Hz) and illumination levels, the combined melanopsin + LMS cone-directed modulation thresholds (Figures 3C, 3E, 4C, and 4E, orange) and slopes (Figures 3D, 3F, 4D, and 4F, orange) are independent of their phase. The combined thresholds are lower than the LMS cone-directed threshold (i.e., cone-pathway facilitation).

In the control rod + cone-directed modulation (1 and 10 Hz), the thresholds and slopes are dependent on their phase at both illuminations (Figures 3B and 4B, blue), with the maximal elevation between 30 and 60° (Figures 3C, 3E, 4C, and 4E), beyond which the psychometric slope is steepest (Figures 3D, 3F, 4D, and 4F). These rod-cone interactions are in line with previous observations (Sun et al., 2001). Next, we determine the nature of the summation between the combined melanopsin and rod or cone signals.

### The summation of melanopsin, rod and cone signals determine visual performance

**Figure 5:**
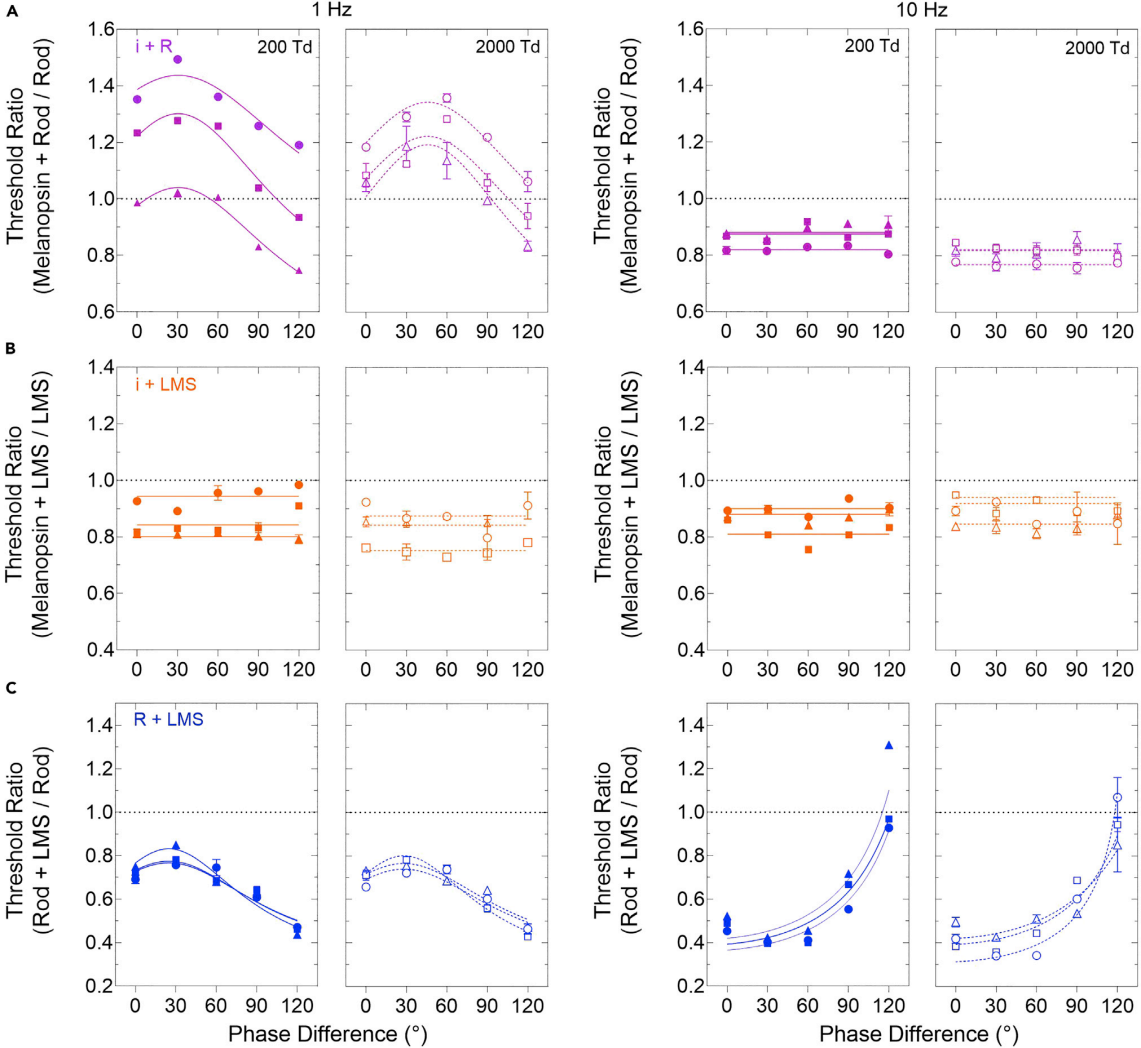
The combination melanopsin (i), rod (R), and cone (LMS) phase summation models Each plot shows a combined photoreceptor to individual photoreceptor-directed threshold ratio (TR) as a function of the relative phase difference for 1 Hz (left panels) or 10 Hz stimulation (right panels) at either 200 Td (filled symbols) or 2000 Td (unfilled symbols). Individual datasets (mean ± SEM) are shown for each of three observers with the best-fitting summation models (Equations 1 and 2), indicated by the solid and dashed lines. The horizontal dotted line (TR = 1) indicates no summation; TRs <1 indicate facilitation and TRs >1 inhibition. (A) Melanopsin + rod-directed modulations (magenta) show phase-dependent, linear summation at 1 Hz and phase-independent, probability summation at 10 Hz. (B) Melanopsin + cone-directed modulations (orange) show probability summation at both temporal frequencies and illumination levels. (C) Rod + cone-directed modulations (blue) follow linear summation.

To evaluate if there is linear or probability summation between the combined melanopsin and rod or cone signals (Figure 3, Figure 4 and 4), we then calculated the threshold ratio (Combined Threshold/Individual Threshold) as a function of phase (Figure 5). At 1 Hz, the melanopsin + rod threshold ratio follows linear summation (Figure 5A; RM-ANOVA; 200 Td, F_4,8_ = 305.80 to 1010.33, p = 0.0001 to 0.0012, *χ*^*2*^_*10*_ *= 14.70 to 15.14,* p *= 0.143 to 0.127*; 2000 Td, F_4,8_ = 12.16 to 25.25, p = 0.025 to 0.031, *χ*^*2*^_*10*_ *= 14.96 to 16.50,* p *= 0.135 to 0.133* in three observers) with the rod signal leading melanopsin by ∼85–∼127 ms. In two of three observers, the melanopsin + rod interaction leads to higher visual thresholds.

At 10 Hz, the threshold ratio (<1.0) is independent of phase (Figure 5A; RM-ANOVA; 200 Td, F^4,8^ = 1.44 to 18.23, p = 0.21 to 0.35, *χ*^*2*^_*10*_ *= 12.98 to 14.13,* p *= 0.22 to 0.167*; 2000 Td, F_4,8_ = 0.24 to 2.08, p = 0.26 to 0.67, *χ*^*2*^_*10*_ *= 14.84 to 15.49,* p *= 0.13 to 0.115* in three observers) and probability summation between the visible rod and perceptually invisible melanopsin signal leads to a facilitation of visual threshold, with the summation index ranging from 0.91 to 2.38 dB.

At both temporal frequencies (1 and 10 Hz), the threshold ratio (<1.0) is independent of phase, and probability summation between the melanopsin + LMS cone signals leads to a facilitation of visual threshold (Figure 5B; RM-ANOVA; 1Hz: 200 Td, F_4,8_ = 0.91 to 12.97, p = 0.06 to 0.45, *χ*^*2*^_*10*_ *= 14.64 to 15.37,* p *= 0.145 to 0.119*; 2000 Td, F_4,8_ = 1.38 to 5.42, p = 0.09 to 0.35, *χ*^*2*^_*10*_ *= 14.37 to 15.12,* p *= 0.156 to 0.12*; 10 Hz: 200 Td, F_4,8_ = 4.25 to 14.95, p = 0.052 to 0.174, *χ*^*2*^_*10*_ *= 16.80 to 17.86,* p *= 0.77 to 0.057*; 2000 Td, F_4,8_ = 1.26 to 9.48, p = 0.08 to 0.37, *χ*^*2*^_*10*_ *= 15.73 to 16.97,* p *= 0.107 to 0.075* in three observers). The summation index ranges from 0.53 to 2.49 dB.

In the control condition, the rod + cone threshold ratio follows linear summation (Figure 5C; RM ANOVA; 1 Hz: 200 Td, F_4, 8_ = 41.70 to 334.81, p = 0.0029 to 0.008, *χ*^*2*^_*10*_ *= 10.81 to 11.71,* p *= 0.372 to 0.304*; 2000 Td, F_4,8_ = 44.91 to 86.33, p = 0.006 to 0.01, *χ*^*2*^_*10*_ *= 13.83 to 16.39,* p *= 0.180 to 0.088*; 10 Hz: 200 Td, F_4, 8_ = 99.0 to 610.8, p = 0.0001 to 0.0092, *χ*^*2*^_*10*_ *= 15.55 to 16.81,* p *= 0.113 to 0.078*; 2000 Td, F_4,8_ = 19.39 to 215.1, p = 0.004 to 0.04, *χ*^*2*^_*10*_ *= 14.94 to 15.86,* p *= 0.134 to 0.103* in three observers) with the cone pathway signal leading the rod signal by between ∼43 and ∼89 ms, with shorter latency differences at the lower frequency (Table 1).

### Melanopsin-rod interactions in daylight depend on their relative response weights to the viewing conditions

**Figure 6:**
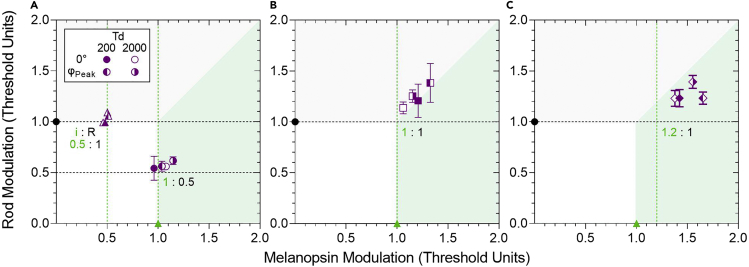
A summation square plot demonstrating contrast-dependent melanopsin-rod interactions measured at a low temporal frequency (1 Hz) within the perceptual resolution limit of the melanopsin pathway The combined rod-directed and melanopsin-directed threshold contrast is normalized to the individual photoreceptor threshold (rods, black circles; melanopsin, green triangles). (A–C) 1.0 Threshold Unit (TU) represents the individual photoreceptor-directed visual threshold; data (mean ± SEM) in the unshaded regions indicate facilitation of the combined threshold (TU < 1), whereas the shaded regions represent inhibition (TU > 1). The diagonal (45°) indicates an equal effect of the interaction on melanopsin and rod thresholds. For data above the diagonal, the interaction causes greater inhibition of the rod pathway; for data below the diagonal, the interaction causes greater inhibition of the melanopsin pathway. The larger the deviation from the initial threshold ratio (dotted horizontal or vertical lines), the greater the inhibition. The panels show the data plotted as a function of threshold contrast ratios that are (A) subthreshold (0.5:1.0 and 1.0:0.5), (B) threshold (1:0:1.0, data from Figure 5), and (C) suprathreshold (1.2:1.0) stimuli at 200 Td (filled symbols) and 2000 Td (unfilled symbols). The combined stimuli are measured in-phase (0° offset) and at their peak interaction phase (30° for 200 Td; right half-filled symbols) and 45° for 2000 Td (left half-filled symbols).

We find that the rod pathway contrast sensitivity is ∼1.25 times higher than the melanopsin pathway at low temporal frequencies (Figure 2A). Nulling these contrast sensitivity differences (1:1 threshold unit; 1 Hz, 0° phase offset) leads to destructive interference of the combined melanopsin + rod signals and an increase in visual threshold (Figure 5A). On the other hand, when progressively higher (subthreshold, in-phase temporal modulation) rod contrasts are added to a melanopsin-directed stimulus, there is a negligible change in melanopsin pathway contrast sensitivity (Figure 2B). We therefore wanted to determine the role of the relative effects of differences in the melanopsin and rod pathway contrast sensitivity and latency on the nature of the low temporal frequency interaction (i.e., suppressive, facilitatory or independent). To do this, we studied the interaction using different melanopsin:rod threshold ratios (subthreshold to suprathreshold: Figure 6A 0.5:1.0, 1.0:0.5, 6(B) 1:0:1.0, or 6(C) 1.2:1.0) at two temporal phase offsets, one in-phase (as per Figure 2B) and the other at 30° or 45° offset to coincide with the offsets causing maximum interference (as per Figure 5A). The threshold measured for the different ratios are plotted in summation squares as a function of the normalized individual photoreceptor threshold (Figure 6). An interaction is present (i.e., suppression or facilitation) when the measured combined threshold data are not aligned to their initial threshold ratio values (vertical and horizontal dashed lines). We do not detect the presence of an interaction when one or the other photoreceptor-directed stimuli is subthreshold, in mesopic or photopic lighting, for either of the measured temporal phase-offsets (melanopsin:rod, 0.5:1 or 1:0.5) (Figure 6A, purple). When melanopsin and rod signals are at threshold (1:1, Figure 6B) or above threshold (1.2:1.0, gamut limit, Figure 6C), an interaction causes the thresholds to increase by 13%–50% (1.13–1.5 threshold units), at both illuminations (Figures 6B and 6C). The melanopsin-rod interaction at lower temporal frequencies (Figure 5A) therefore depends on both their relative phase and sensitivity to the stimulus contrast.

## Discussion

Extending to high photopic illuminations, we observe that the melanopsin pathway has the lowest contrast sensitivity of all photoreceptor pathways (Figure 2) and interacts to alter rod- and cone-mediated vision (Figure 2, Figure 3, Figure 4, Figure 5, Figure 6). These interactions are frequency-dependent and manifest as either linear or probability summation (Figure 5), pointing toward multiple sites of interaction within the visual system. This is in part related to rods escaping saturation up to at least 8000 Ph Td (20000 Sc Td) (Figures 2G and 2H), with their temporal sensitivity in photopic illumination following a frequency-dependent, bipartite process with greater attenuation at higher (Figure 2H) than lower temporal frequencies (Figure 2G). When subthreshold, the rod pathway can intrude in melanopsin-directed responses with as little as ∼3% rod contrast (Figure 2B). A similar effect is evident with low-contrast cone signals intruding in rod-directed stimuli (Figure 2C). These findings have direct implications for determining how and where in the visual system the rod and cone signals combine with melanopsin signals to drive human temporal contrast sensitivity, and in the evaluation and interpretation of data from silent-substitution paradigms applied to measure melanopsin-directed visual and nonvisual functions and their interrelations with the rod and cone pathways. With five photoreceptor classes in human eyes, any application of multi-primary optical photostimulators therefore requires a direct test of the assumption that rods are saturated for the viewing conditions typically used in the experiments (e.g., ∼2300 to 14500 Ph Td; 70 to 90% rhodopsin pigment available) (Agrici et al., 2019; Allen et al., 2019; Brown et al., 2012; DeLawyer et al., 2020; Spitschan et al., 2017; Vincent et al., 2021), determining the type of interaction or establishing that no interactions exist between the rod and melanopsin pathways for the spatiotemporal stimulation and illumination level.

The transition between inhibitory linear summation and facilitatory probability summation of the melanopsin and rod signals is related to the melanopsin pathway temporal sensitivity (Figure 5A). Inhibition of contrast sensitivity occurs when both the melanopsin and rod signals are at or above threshold (Figure 6) and nearer the peak of the melanopsin pathway temporal resolution (e.g., 1 Hz). Because the intrinsic melanopsin response is mediated exclusively via ipRGCs in primates (Dacey et al., 2005; Grünert and Martin, 2021; Jusuf et al., 2007; Liao et al., 2016), whereas rod signals input to multiple pathways, including extrinsically to ipRGCs (Hannibal et al., 2017; Patterson et al., 2020) and the canonical retinogeniculate pathways that also support cone vision (Cao et al., 2010; Field et al., 2009; Gouras and Link, 1966; Lee et al., 1997; Virsu et al., 1987), the inhibitory interaction could involve intraretinal pathways, cortical circuits, or their combination. Intraretinal networks including VGlut3 amacrine cells can send inhibitory feedback in primates (Marshak et al., 2015; Patterson et al., 2020) but these are undefined for the melanopsin pathway. At a systems level, we show that rod signals lead the melanopsin signals by ∼85–127 ms (Figure 5A), hence the timing of the peak interaction is sufficiently long to invoke cortical sites given that signals reach the primate cortex within 66 ± 11 ms (Schmolesky et al., 1998). The latency difference increases with illumination (Figure 5A) because of the relative stability of the melanopsin pathway temporal contrast response compared to the progressively more band-pass response of the rod pathway (Figure 5A). Further work will be required to understand the factors that set these latencies and the interactions.

The high frequency interaction means that melanopsin stimulation does not need to be perceptually visible to interact with and alter rod and cone-mediated functions. Beyond the resolution limit of melanopsin vision, probability summation between the melanopsin and rod or cone pathways facilitates their contrast sensitivity (Figures 5A and 5B) with the summation index ranging from 0.9 to 2.15 dB. Probability summation is also present at lower temporal frequencies, including subthreshold facilitatory interactions at frequencies (≥3 Hz) in the proximity of the peak rod temporal contrast response (Figure 2C). Previously, higher melanopsin excitation levels were shown to enhance cone-mediated visual contrast discrimination (Zele et al., 2019b) but not under viewing conditions wherein rod excitations were left uncontrolled (Vincent et al., 2021). This indicates the relative response properties of all three pathways and the weights of the inhibitory and excitatory interactions occurring in the observational conditions are critical for setting visual contrast sensitivity. These interactions also have consequences for the development of vision tests designed to detect subtle visual contrast loss in disease when there is melanopsin dysfunction (Chougule et al., 2019; Feigl et al., 2011, 2012; Joyce et al., 2018; La Morgia et al., 2016; Maynard et al., 2015; Munch et al., 2015; Najjar et al., 2018; Park et al., 2017) and thus an associated change in its support of rod-mediated and cone-mediated vision in retinal disease. Facilitatory summation could involve at least two intraretinal excitatory pathways; one involving ipRGC to RGC connections via glutamatergic amacrine cells as identified in primates (Marshak et al., 2015; Patterson et al., 2020) and another ipRGC to outer retinal connection via dopaminergic amacrine cells as identified in mice (Newkirk et al., 2013; Zhang et al., 2008). Given the temporal lead of the cone pathways, the summation with melanopsin could also occur later in the visual system at a cortical locus.

The sensitivity of rod-mediated and cone-mediated visual functions reflect the spectral, spatiotemporal, and adaptation properties and viewing eccentricity of the measurement conditions (Barlow, 1957; Hecht and Smith, 1936), factors presently less well-examined for melanopsin photoreception. For the few studies using different methodological paradigms to quantify threshold level melanopsin-directed visual function, the visual contrast thresholds are in the range of 10%–15% Michelson contrast with periodic stimuli (Allen et al., 2019; Cao et al., 2015; Horiguchi et al., 2013; Zele et al., 2018b) and similarly, about 12%–16% Weber contrast (Zele et al., 2018b, 2019b), as per the range of melanopsin thresholds observed here (Figure 4, Figure 5A and 5A). In comparison to the absolute dark-adapted sensitivity of the rod- and cone-pathways, melanopsin contributions to vision require illuminations ∼200 time higher (Zele et al., 2019b) than necessary at the limits of cone vision (∼1 photopic Td) and ∼2 million times higher than the rod pathway at the absolute threshold of vision (∼9.3 × 10^−5^ photopic Td) (Dey et al., 2021; Hecht et al., 1942). Our data therefore have implications for the development of a triplicity theory of mesopic and photopic vision.

The bipartite change in the rod pathway temporal resolution at ∼3000 Ph Td (7500 Sc Td) drives its high frequency response down to within its mesopic limits while retaining high contrast sensitivity at low temporal frequencies (Figure 2H). However, rod-mediated vision is still possible because at least 78% of the rhodopsin photopigment is still available for photon capture at our highest illumination (Rushton and Powell, 1972; Thomas and Lamb, 1999). A transition to the cone pathway cannot explain the robust photopic rod temporal response measured in our silent-substitution protocol, because we eliminated cone signal artefacts from rod-directed stimuli (Figure 2H). Moreover, if an artifact was present in the silent substitution, it would manifest at all temporal frequencies, but this is not the case. During the transition between rod-mediated and cone-mediated vision, it is known that rod pathway sensitivity is dependent on post-receptoral interactions wherein higher L-cone excitations result in a steeper Threshold versus Intensity (TvI) function (Shapiro, 2002; Sharpe et al., 1989a), consistent with our experimental conditions wherein the orange-appearing adapting background has a higher L-cone excitation level than an equal energy white spectrum. In mesopic lighting, the rod suppression of cone-mediated vision (Alexander and Fishman, 1984; Cao and Lu, 2012; Cao et al., 2006; Coletta and Adams, 1984; Goldberg et al., 1983) acts to reduce the latency differences between the two systems by inhibiting the cone pathway temporal response, thereby improving visual processing in twilight conditions (Zele et al., 2008). Here, we reveal a parallel process in photopic lighting wherein the rod, cone, and melanopsin pathways operate collectively and interact via both inhibitory (Figure 5A) and facilitatory mechanisms (Figures 5A and 5B) to regulate daylight temporal visual performance.

### Limitations of the study

Our interaction estimates are based on the visibility of threshold-equated melanopsin-directed, rod-directed, and cone-directed stimuli at a reference chromaticity with fixed ratio photoreceptor excitations; it remains to be evaluated how the nature and magnitude of the interaction depends on these excitation ratios. When melanopsin sensitivity to the stimulus modulation shifts from perceptually visible to invisible, the melanopsin-rod interaction transitions from inhibitory to facilitatory threshold summation. However, the interaction pattern within the transition region was not evaluated and might reveal destructive interference and perhaps cancellation because of differences in the temporal phase characteristics and adaptation behavior of the photoreceptor signals. Such interactions could together set visual contrast sensitivity in viewing conditions when all photoreceptor classes jointly input to the resultant signal.

## STAR★Methods

### Key resources table

**Table:** 

REAGENT or RESOURCE	SOURCE	IDENTIFIER
**Deposited data**		

QUT Research Data Finder	Queensland University of Technology (QUT)	https://doi.org/10.25912/RDF_1645061980220

**Software and algorithms**		

GraphPad Prism 9	GraphPad Software	https://www.graphpad.com/scientific-software/prism/
IBM-SPSS v25.0	International Business Machines Corporation	https://www.ibm.com/analytics/spssstatistics-software

**Other**		

Apple MacPro QuadCore Intel computer	Apple, Inc.	https://www.apple.com/mac/
Arduino Uno SMD R3, Model A000073	Arduino	https://www.arduino.cc/
EPP2000C-50um Slit UV-VIS Spectrometer	StellarNet	https://www.stellarnet.us
ILT1700Research Radiometer	International Light Technologies, Inc.	https://www.intl-lighttech.com/products/ilt1700-research-radiometer

### Resource availability

#### Lead contact

Further information and requests for resources should be directed to and will be fulfilled by the lead contact, Professor Andrew J. Zele (andrew.zele@qut.edu.au).

#### Materials availability

This study did not generate new unique reagents.

#### Data and code availability

• De-identified human data have been deposited at the QUT Research Data Finder. They are publicly available as of the date of publication. DOIs are listed in the key resources table.
• This paper does not report original code.
• Any additional information required to reanalyse the data reported in this paper is available from the lead contact upon request.

### Experimental model and subject details

All experimental protocols were conducted in accordance with a Queensland University of Technology (QUT) Human Research Ethics Committee approval (no. 1700000510) and followed the tenets of the Declaration of Helsinki; written informed consent was obtained from all participants. Three healthy observers (all males; O1, 31 years; O2, 29 years; and O3, 38 years) participated in the study who had trichromatic color vision (Ishihara pseudoisochromatic plates and L’anthony Desaturated D-15 Test), visual acuity of 0.0 logMAR (6/6) or better, age-normal spatial contrast sensitivity (Combined Spatial Contrast and Visual Acuity chart (Adhikari et al., 2022), no ocular diseases as confirmed with ophthalmoscopy, fundus photography (Canon Non Mydriatic Retinal Camera, CR-DGi, Canon Inc., Tokyo, Japan), optical coherence topography (RS-3000 OCT RetinaScan Advance, Nidek Co., Ltd., Tokyo, Japan) and intraocular pressure measurement (<21 mmHg) (Icare® ic100; Icare Finland Oy, Vantaa, Finland), and no systemic disease. Two observers were authors, and one was an experienced observer who was naïve to the purpose of the experiment. The small sample size is justified based on high repeatability of human psychophysical data and the extensive experimental protocol involving a total testing time (excluding dark adaptation) of ∼120 h per observer.

### Method details

#### Apparatus and calibration

Stimuli were generated using a custom-built 5-primary photostimulator (Cao et al., 2015) with light emitting diode and interference filter combinations (Ealing, Natick, MA, USA) producing primary lights of peak wavelengths (± full width at half maximum) at 456 nm (10 nm), 488 nm (11 nm), 540 nm (10 nm), 594 nm (14 nm), and 633 nm (15 nm). The pulse width modulation can generate stimuli with frequencies up to ∼488 Hz at 12-bit resolution per primary light (Cao et al., 2015). Zele et al. (2018b) provides details of the photostimulator calibration. Photoreceptor excitations were calculated based on the CIE 1964 10° Standard Observer cone fundamentals (Smith and Pokorny, 1975), the CIE 1951 scotopic luminosity function, and the melanopsin spectral sensitivity function (Enezi et al., 2011). This CIE 1964 10° Standard Observer represents the spectral luminous efficiency of an ideal observer viewing larger stimulus fields that extend into the parafoveal retina. Retinal illumination (photopic) was specified as the sum of L- and M−cone excitations with a 2:1 L:M cone ratio (MacLeod and Boynton, 1979), such that for an equal energy spectrum at 1 photopic Troland (Td), the photoreceptor excitation relative to photopic illumination was 0.6667 for L-cones (*l*), 0.3333 for M-cones (*m*), 1 for S-cones (*s*), 1 for rods (*r*), and 1 for melanopsin (*i*). All Troland values reported hereafter are photopic (Ph Td) unless specified as scotopic (Sc Td). We maximised the instrument gamut using an orangish appearing adapting background having relative photoreceptor excitations of *s* = 0.107, *m* = 0.244, *l* = 0.755, *r* = 0.345, and *i* = 0.265. To account for individual differences in pre-receptoral filtering and photoreceptor spectral sensitivities between the observer and the CIE 1964 10° standard observer, participants performed heterochromatic flicker photometry (HFP) at 25 Hz using a rectangular wave counterphase flicker between a reference (green) and each of the test primaries (blue, cyan, amber, and red) superimposed on a 20 Td neutral white adapting background (CIE 1964 10° *x, y* = 0.3318, 0.3857) to produce a time-averaged illumination of 30.3 Td (see Uprety et al., 2021 for details of the HFP procedure). The HFP outputs were applied to scale the theoretical 10° standard observer data (for details, see Zele et al., 2019b; Uprety et al., 2021).

#### Experimental design

##### General stimulus specifications

Silent substitution (Estevez and Spekreijse, 1982; Shapiro et al., 1996) was used to specify the mixtures of five physical primary lights (Cao et al., 2015) required to independently control the five photoreceptor excitations. By doing so, up to four photoreceptor classes can be silenced to allow investigation of the functional properties of a fifth class, or any combination of photoreceptor classes (Gnyawali et al., 2022). Stimuli were presented in Maxwellian view as a uniform annular field of 30° outer diameter and 10.5° inner diameter (Cao et al., 2015). The centre of the black, 10.5° diameter macular blocker contained the fixation marker. Six photoreceptor-directed stimulus combinations were implemented, including three photoreceptor-specific and another three combination-stimuli designed to study photoreceptor interactions: (1) Intrinsic melanopsin-directed (i) stimuli with no change in the excitation of rhodopsin and three cone opsins; (2) rhodopsin-directed (R) stimuli with no change in the excitation of melanopsin and the three cone opsins; (3) L-, M− and S-cones modulated in-phase to produce cone luminance- (+L + M + S) directed stimuli with no change in the excitation of rhodopsin and melanopsin (i.e., cone-directed stimuli); (4) combined i and R directed stimuli (with constant LMS excitation); (5) combined i and LMS directed stimuli (with constant R excitation); and (6) combined R and LMS directed stimuli (with constant i excitation).

The test stimuli were sinusoidal temporal modulations (0.2–70 Hz). We opted for flickering stimuli because the phase relationship between the combined photoreceptor-directed stimuli can be controlled under a constant, time-averaged state of adaptation (Kremers and Meierkord, 1999; Sun et al., 2001) to directly assess the effects of physiological differences in the temporal responses of the photoreceptors and their associated pathways (Davis et al., 2015; Do et al., 2009; MacLeod, 1972; Sharpe et al., 1989b; Van den Berg and Spekreijse, 1977; Zele et al., 2008). For frequencies ≥1 Hz, the stimulus duration was 1000 ms; for frequencies <1 Hz, the duration was the reciprocal of the frequency. Key experimental conditions applied two temporal frequencies; at 1 Hz near the peak melanopsin temporal contrast response (Allen et al., 2019; Zele et al., 2018b) and at 10 Hz, beyond melanopsin pathway temporal resolution. Flicker detection thresholds were estimated using a method of adjustment to change the temporal frequency in 1 or 5 Hz steps at a fixed photoreceptor-directed contrast. A 2000 ms pre- and post-stimulus period included photoreceptor-directed temporal white noise (TWN) that randomly modulated the *s m l r* photoreceptor excitations (40% Michelson contrast) (Hathibelagal et al., 2016, 2018) without changing the melanopsin (i) excitation (Zele et al., 2018b, 2019b). For the open-field melanopsin-directed stimulation condition, the rod contrast was ≤0.3%; for the rod isolating condition, the LMS-cone contrast was ≤1.5% (for calculations, see Zele et al., 2018b).

The mean adaptation levels spanned 0.2–8000 Ph Td (0.5–19920 Sc Td) with key experimental conditions conducted in mesopic (200 Td, ∼14.09 log quanta.cm^−2^.s^−1^) and photopic illumination (2000 Td, ∼15.11 log quanta.cm^−2^.s^−1^). All participants completed individual observer calibrations (e.g., HFP, color matching and bleach recovery; detailed in Zele et al., 2018b) before starting the experiments (Uprety et al., 2021).

##### Psychophysical paradigms

To define the tolerance limits of the silent-substitution, we first evaluated visual temporal contrast sensitivity to melanopsin- and rhodopsin-directed stimulation with signal artefacts introduced from unmodulated photoreceptors by adding variable levels of supplemental rhodopsin- or cone-opsin contrast (e.g., i + ΔR; R + ΔLMS). If there is significant rod intrusion in melanopsin-directed stimuli, or cone intrusion in rod-directed stimuli, the peak of the measured TCSF and critical flicker frequency (CFF: Measured at LMS = 28%, rod = 15% and melanopsin = 17% Michelson contrast) will shift toward the more sensitive rod- or cone-mediated temporal contrast response, beyond the resolution of their respective photoreceptor-isolated temporal capability. The absolute amplitude sensitivity as a function of the photoreceptor modulation frequency was described with a difference of Gaussians function (Enroth-Cugell and Robson, 1966). A Levenberg–Marquardt algorithm minimised the sum of squared differences between the model and data by varying three free parameters (amplitude, mean sensitivity, and the SD). Temporal white noise was applied to eliminate the cone intrusion in melanopsin-directed stimuli (Zele et al., 2019a; b; Zele et al., 2018b).

To determine the dependence of the melanopsin, rod and cone photoreceptor interactions on their temporal phase differences, we then implemented a phase summation paradigm (Sun et al., 2001; Zele et al., 2012). The Michelson contrast at threshold (63.21% yes responses) for each photoreceptor-directed stimulus condition was initially estimated from the best-fitting Weibull psychometric functions describing observer performance (i.e., frequency of seeing data) as a function of stimulus contrast with a method of constant stimuli. The stimuli were scaled in multiples of the observer’s detection threshold (i.e., threshold units) to compare performance initiated by the different photoreceptor classes. For example, if the melanopsin-directed detection threshold is 10% and the rod-directed detection threshold is 8%, then for a 1:1 threshold unit (TU), these photoreceptor conditions are combined as 10% i + 8% R. This approach was used to determine the contrast range for the combined photoreceptor conditions, such that the relative amplitude of the two photoreceptor modulations was always in a ratio of 1:1 (TU = 1:1). The abscissa of the psychometric functions included rod contrast for the i + R and R + LMS combinations and LMS-cone contrast for the i + LMS combination. The Weibull model fit was optimised by varying two free parameters (threshold and slope), with the lapse rate fixed at zero and the guessing rate was corrected prior to fitting the model (Hathibelagal et al., 2016). These threshold values were periodically re-tested during the experiments to ensure their stability. The phase data measured as a function of the ratio of the combined to individual photoreceptor threshold [(Combined Threshold)/Individual Threshold = (*i* + *r*)_Threshold_/r_Threshold_ or (*i* + *c*)_Threshold_/*c*_Threshold_)] were then modeled as probability summation if threshold was independent of phase, or as linear summation if the threshold was dependent on the relative phase difference of the combined photoreceptor-directed flicker. For probability summation, the threshold ratios (TR) can be described by a linear function parallel to abscissa,

Equation 1 TR=n

where *n* is a free vertical scaling factor (Figure 1, dashed line) representing the summation index (*n* in linear units = 20∗log_10_(^1^/_*n*_) dB; (Graham, 1989). For linear summation (Figure 1, solid lines), a vector summation model (Van den Berg and Spekreijse, 1977) was applied where the threshold response, *V*_*t*_, to the combined photoreceptor-directed modulation (e.g., melanopsin, i and rod, *r*, modulations) was calculated as,

Equation 2 $$V_{t}=\frac{1}{\sqrt{({\text{r}}^{2}+{\text{ i}}^{2}-2.\text{ r }.\text{ i }.\text{ cos}\left(\text{φ}-{\text{φ}}_{\text{r}-\text{i}}\right))}}$$

where φ is the stimulus phase (0° to 120°) and φ_r-i_ is the temporal phase difference between the photoreceptor signals. The precision of the estimate of the temporal phase difference was increased by describing all observer datasets with one phase parameter (e.g., φ_r-i_) (a global phase estimate), with the assumption that the same photoreceptor-directed response measured under the same adaptation condition is mediated by the same mechanism in all observers. The photoreceptor-directed modulation sensitivity parameter was varied separately for each observer to account for individual sensitivity differences. A Levenberg–Marquardt algorithm minimised the sum of square differences between the model and data by varying the free parameters (e.g., melanopsin, i, and rod, r photoreceptor-directed modulations; temporal phase difference, φ_r-i_). The relative effects of differences in the melanopsin and rod pathway contrast sensitivity and latency on low temporal frequency interactions were further explored by introducing a contrast paradigm based on the outcomes of the phase paradigm experiment performed with 1:1 threshold units and the temporal contrast sensitivity experiment. To test the hypothesis that melanopsin-rhodopsin interaction is affected by the relative weight of their individual photoreceptor contrast thresholds, the contrast paradigm evaluated detection thresholds for the combined stimuli at sub-threshold (i:R = 0.5:1.0, 1:0.5), threshold (1:1, data from the phase paradigm experiment) and suprathreshold (1.2:1) contrast ratios. In the resultant summation-square plot, rod-directed thresholds are shown as a function of the melanopsin-directed thresholds in threshold units for the different threshold ratios.

#### General procedure

Testing sessions were completed by observers at a similar time each day to limit any effect of circadian variation on melanopsin-mediated function (Zele et al., 2011). Before an experiment, observers were habitually exposed to ∼1 h of indoor artificial lighting (luminance, ∼100 cd m^−2^; illuminance, ∼230 lux) that produced 3.7% rhodopsin bleach (Rushton and Powell, 1972; Thomas and Lamb, 1999). Following a further 15 min dark adaptation period prior to data collection, rhodopsin bleach levels were estimated at 0.3%, lower than the rhodopsin bleach level with the 200 Td (0.6% rhodopsin bleach) and 2000 Td (6.0% rhodopsin bleach) adaptation levels. Melanopsin adaptation is 1.2 times faster than rhodopsin but 3.4 times slower than the cone-opsins (Pant et al., 2021). Pilot data showed no significant difference in contrast threshold with longer dark adaptation periods (up to 30 min). For adaptation levels ≤20 Td, the dark adaptation duration was 30 min. Psychophysical paradigms began after a 2 min light adaptation to the steady illumination level of the background adapting stimulus. Observer responses were recorded using a hand-held gamepad. Each observer participated in 159 testing sessions, with each session lasting ∼45 min excluding dark adaptation (15 or 30 min depending on light level). This included 64 sessions for the temporal contrast sensitivity (TCS) experiments, 84 sessions for the phase summation experiments, and 11 sessions for the critical flicker frequency (CFF) experiments. For each testing condition, each observer completed at least three repeated measurements for the TCS and summation experiments and at least 10 repeated measurements for the CFF experiments. Breaks were provided where required during a testing session to minimise fatigue. Estimation of frequency of seeing functions with the method of constant stimuli included 24 trials (80% probability) plus 6 catch trails (20% probability) for each of the 6-7 contrast levels. For each psychophysical paradigm, the conditions (e.g., photoreceptor-directed stimulus, phase, and frequency) were randomised across sessions. When light level was an independent parameter, we always started from the dimmest light level and then increased stepwise to avoid cumbersome re-adaptation periods before starting a new light level (Aguilar and Stiles, 1954; Hecht and Shlaer, 1936).

#### Photoreceptor interaction models

Model predictions (no-interaction, linear or probability summation) for the phase summation paradigm are presented in Figure 1. At ∼200 Td, the latency to peak temporal response is estimated at ∼35 ms for the cone pathway (Cao et al., 2007) and ∼80 ms for the rod pathway (Maguire et al., 2016; Zele and Cao, 2014) as determined using human psychophysical data and ERGs, and at ∼250 ms for melanopsin cells as evident in *in-vivo* recordings (Do et al., 2009). These temporal response latency differences were used for the initial model predictions shown in Figure 1.

### Quantification and statistical analysis

Statistical analyses were conducted using the statistical package SPSS (version 25.0, SPSS Inc, Chicago, Illinois, USA) and GraphPad Prism (GraphPad Software, Inc., CA, USA). The data frequency distributions were estimated using the D’Agostino and Pearson omnibus normality test. A one-way repeated measures ANOVA (Mauchly’s test for data sphericity, 95% confidence interval, p < 0.05, post-hoc Bonferroni correction) was performed to determine the effect of supplemental rod or cone intrusion on melanopsin or rod contrast sensitivity and to determine if the threshold ratio was dependent on the phase offset of the combined photoreceptor directed stimuli. If the threshold ratio was independent of phase, the data were described using probability summation (Equation 1) otherwise, linear summation (Equation 2). The goodness of fit of the model to the data was assessed using a Chi-square test (p > 0.05).
